# Characterization and Endocytic Internalization of Epith-2 Cell Surface Glycoprotein during the Epithelial-to-Mesenchymal Transition in Sea Urchin Embryos

**DOI:** 10.3389/fendo.2013.00112

**Published:** 2013-08-30

**Authors:** Norio Wakayama, Tomoko Katow, Hideki Katow

**Affiliations:** ^1^Research Center for Marine Biology, Tohoku University, Aomori, Aomori, Japan

**Keywords:** Epith-2, EMT, cell surface modification, protein tyrosine kinase, growth factor receptor, sea urchin

## Abstract

The epithelial cells of the sea urchin *Hemicentrotus pulcherrimus* embryo express an Epith-2, uncharacterized glycoprotein, on the lateral surface. Here, we describe internalization of Epith-2 during mesenchyme formation through the epithelial-to-mesenchymal transition (EMT). Epith-2 was first expressed on the entire egg surface soon after fertilization and on the blastomeres until the 4-cell stage, but was localized to the lateral surface of epithelial cells at and after the 16-cell stage throughout the later developmental period. However, primary mesenchyme cells (PMC) and secondary mesenchyme cells (SMC) that ingress by EMT lost Epith-2 from their cell surface by endocytosis during dissociation from the epithelium, which was associated with the appearance of cytoplasmic Epith-2 dots. The cytoplasmic Epith-2 retained a similar relative molecular mass to that of the cell surface immediately after ingression through the early period of the spreading to single cells. Then, Epith-2 was completely lost from the cytoplasm. Tyrosine residues of Epith-2 were phosphorylated. The endocytic retraction of Epith-2 was inhibited by herbimycin A (HA), a protein tyrosine kinase (PTK) inhibitor, and suramin, a growth factor receptor (GFR) inhibitor, suggesting the involvement of the GFR/PTK (GP) signaling pathway. These two GP inhibitors also inhibited PMC and SMC spreading to individual cells after ingression, but the dissociation of PMC and SMC from the epithelium was not inhibited. In suramin-treated embryos, dissociated mesenchyme cells migrated partially by retaining their epithelial morphology. In HA-treated embryos, no mesenchyme cells migrated. Thus, the EMT occurs in relation to internalization of Epith-2 from presumptive PMC and SMC.

## Introduction

The epithelial-to-mesenchymal transition (EMT) occurs in various processes found in metazoans, such as normal morphogenesis, the mesoderm formation in rabbit embryos (1), and neural crest cell formation in vertebrates [rev. Ref. (2)]. The EMT also constitutes the basic mechanism of metastasis (3, 4) and endocrine system formation, including the shedding of human fetal pancreatic insulin-producing cells from pancreatic islets (5) and luteinizing hormone-releasing hormone-immunoreactive neurons from the placodal epithelium (6).

In invertebrates, the EMT has been reported in relation to mesenchyme formation, particularly in sea urchin embryos in which the ingression of primary mesenchyme cells (PMC) occurs at the vegetal plate region of late blastulae (7–10). The EMT also produces secondary mesenchyme cells (SMC) at the tip of the archenteron in late gastrulae (8, 10, 11). The EMT is associated with an alteration of cell surface properties such as the retraction of epithelial cell surface-specific Epith-1 protein that is recognized by a monoclonal antibody (mAb), anti-Epith-1mAb, in the sea urchin *Temnopleurus hardwicki* (10), and the acquisition of PMC surface-specific proteins such as the msp130 protein (12) in the sea urchin *Strongylocentrotus purpuratus* or the P4 protein that is recognized by anti-P4 mAb, in the sea urchin *Hemicentrotus pulcherrimus* (13–15). The EMT is also associated with losing integrin alphaSU2 and an affinity to laminin in the sea urchin *Lytechinus variegatus* (16). Similarly in vertebrate embryos, the EMT occurs by losing epithelial cell marker molecules such as E-cadherin, and the resultant mesenchyme cells acquire mesenchymal cell marker molecules such as vimentin, fibronectin, and type 1 collagen (17, 18).

In the sea urchin *L. variegatus*, β-catenin (19) and cadherin [LvG-cadherin; (20)] are expressed as a complex of these two proteins on the lateral surface of embryonic epithelial cells, particularly near adherence junctions that are dissolved during the early moments of PMC ingression in *L. pictus* (9). These proteins are lost by endocytosis, which results in the dissociation of PMC and SMC from neighboring epithelial cells (19, 20). Whether a similar mechanism is involved in losing cell surface Epith-1 in *T. hardwicki* has remained question (10). Our previous reports indicate that protein tyrosine kinase (PTK) is involved in PMC spreading after the ingression in *H. pulcherrimus* and *Clypeaster japonicus* (21, 22) as well as the retraction of Epith-1 and PMC spreading in *T. hardwicki* (23). PTK signaling pathways also regulate endocytosis (24); therefore, it has been predicted that the retraction of Epith-1 also occurs by endocytosis.

Thus, the present study aimed to elucidate the involvement of endocytosis in losing Epith-2, an epithelial cell surface-specific protein that is recognized by an anti-Epith-2 mAb and its sister mAb, anti-Epith-1 mAb (10), from the epithelial cell surface during PMC ingression. To this end, the experiments used PMCs purified from mesenchyme blastulae using an immunoaffinity column that fixed the magnet-tagged antibody (Ab) against anti-P4 mAb (13, 14), which is specific to PMCs. The potential involvement of PTK was examined using pharmaceutical inhibitors that included the closely related growth factor receptor (GFR) inhibitor. The previous analysis of the epitopic property of the anti-Epith-2 mAb proved that the mAb is an excellent tool to analyze the mechanism of cell surface modification and the function of Epith-2/Epith-1 protein as a cell adhesion molecule instead of the anti-Epith-1 mAb.

## Materials and Methods

Gametes from the sea urchin *H. pulcherrimus*, *T. hardwicki, S. intermedius, Mespilia globules, C. japonicus, L. pictus*, and *Pseudocentrotus depressus* were obtained by blastocoelic injection of 0.5 M KCl. The inseminated eggs were incubated in filtered sea water (FSW) on a gyratory shaker at 15°C for *H. pulcherrimus* and *S. intermedius*, 17°C for *L. pictus*, 18°C for *T. hardwicki* and *C. japonicus*, 19–25°C for *M. globules*, and 20°C for *P. depressus* until the stage described in the text. The majority of the present study was conducted using *H. pulcherrimus*. The *H. pulcherrimus* zygotes and embryos were collected at 20 min post fertilization (fertilized eggs), at 2 h post fertilization (2-hpf) (2-cells), at 2.5-hpf (4-cells), at 5-hpf (16-cells), at 8-hpf (morula), at 16-hpf (swimming blastula), at 19-hpf (mesenchyme blastula), at 23-hpf (1/2 gastrula, gastrulation half completed), at 25-hpf (late gastrula, gastrulation completed), at 29-hpf (prism), and at 40-hpf (pluteus stages). The *T. hardwicki* embryos were collected at 12-hpf (swimming blastula) and at 14-hpf (mesenchyme blastula).

### Immunoblotting

The *H. pulcherrimus* and *T. hardwicki* embryos reached the developmental stages described above, and they along with the *S. intermedius* mesenchyme blastulae, the *M. globules, C. japonicus*, and *L. pictus* swimming blastulae, and the *P. depressus* gastrulae were solubilized in lysis buffer (6 M urea, 1% Nonidet P-40, 10 mM Tris-HCl, pH 7.6) and were precipitated in 70% ethanol at −30°C overnight. The samples were lyophilized, dissolved in 2× sample buffer of sodium-dodecyl sulfate acrylamide gel electrophoresis (SDS-PAGE) with or without 2-mercaptoethanol at 500 μg/ml, separated on SDS-PAGE slab gels, and transferred to nitrocellulose filters (Schleicher Schuell, Dassel, Germany) at 400 mA at 4°C for 2 h as previously described (10). The protein-blotted nitrocellulose filters were blocked with 5% bovine serum albumin (BSA, Sigma Chemical Co. St. Louis, MO, USA) or 10% skim milk (Snow Brand Co. Sapporo, Japan) in TBST (25 mM Tris at pH 7.5, 7.5 mM NaCl, 0.025% Tween-20) for 1 h. The blots were probed with anti-Epith-1 mAb or anti-Epith-2 mAb [Ref. (10); diluted at 1:1000 for anti-Epith-1 mAb and 1:100 for anti-Epith-2 mAb in TBST] by incubating for 2 h at ambient temperature. The primary antibodies were detected with alkaline phosphatase-labeled sheep anti-mouse IgG antibodies (Promega, Madison, WI, USA) diluted at 1:7500 in TBST and were visualized with nitroblue tetrazolium/5-bromo-4-chloro-3-indolyl phosphate (Promega) according to the instructions by the manufacturer.

### Isoelectric points and molecular mass in Dalton separation (ISO-DALT 2D gel)

The lyophilized swimming blastulae of *H. pulcherrimus* and *T. hardwicki* were dissolved in isoelectric focusing sample buffer [9 M urea, 4% Nonidet P-40, and 2% ampholytes (Sigma Chem. Co.); pH 4–7: pH 3.5–10 = 3:1 (25)] at 1 mg/ml. Undissolved debris in the sample was removed by centrifugation at 10,000 × *g* for 15 min. Fifteen microliters of supernatant was loaded onto each isoelectric focusing gel column [9 M urea, 3.15% acrylamide, 0.6% *N*-*N*′methylene bis acrylamide, 2.05% Nonidet P-40, 5% ampholytes (pH 4–7: pH 3.5–10 = 3:1), 0.038% ammonium peroxydisulfate, 0.063% *N*,*N*,*N*″,*N*″-tetramethylethylenediamine] overlayed with 5 μl of sample overlay solution [8 M urea, 2% ampholytes (pH 4–7: pH 3.5–10 = 3:1)], electrophoresed for 3 h at 400 V and then electrophoresed for 30 min at 500 V as for first dimensional gels. The column gels were equilibrated in an equilibration buffer (0.125 M Tris, 2% SDS, 10% glycerol, pH 6.8) for 30 min with constant agitation and were embedded in agarose gel (0.125 M Tris, 0.5% agarose, 0.1% SDS) on the top of the second dimensional slab gel. The second electrophoresis step was conducted at 25 mA, and the separated proteins were visualized by silver stain using a Silver Stain Plus kit (Bio-Rad, CA, USA). Aliquot gels were analyzed with immunoblotting as described above.

The pH gradient was examined using 5-mm long slices cut from a first dimensional column after initial isoelectric focusing. The ampholytes in the gel slices were eluted by immersing these slices in distilled water with agitation for 1 h. The pH of each eluted ampholyte was examined using a MP220 pH meter (Mettler Toledo International Inc., Tokyo) and the entire pH gradient was calculated in an 8-cm long column.

### Detergent extraction of Epith-2

To examine whether the epitope of the anti-Epith-2 mAb is embedded in the plasma membrane similar to the epitope of anti-Epith-1 mAb (10), lyophilized *H. pulcherrimus* swimming blastulae were dissolved in a mixture of 0.1 M Tris, 1% Triton X-100, and 1% glycerol at 1 mg/ml as a detergent soluble fraction. The sample aliquots were dissolved in plain distilled water as a water-soluble fraction, both samples were centrifuged at 10,000 × *g* for 15 min and the supernatant was diluted in 2× SDS-PAGE sample buffer to separate on the slab gels as described above. The separated proteins were transferred to the nitrocellulose filters as described above and used for immunoblotting.

### Concanavalin a binding sites

To detect α-D-mannosyl and α-D-glucosyl groups that are abundant in sea urchin embryos (26), concanavalin A (Con A; Sigma Chem. Co.) was applied to the lyophilized *H. pulcherrimus* swimming blastulae after separation on 10% SDS-PAGE slab gels. The gels were fixed overnight in a mixture of 25% isopropanol and 10% acetic acid and were washed 17 h with 0.1 M phosphate-buffered saline (PBS) by replacing several times with fresh PBS. The samples were incubated with 0.5 mg/ml Con A for 2 h, washed with PBS for 17 h as described above and were incubated with 50 μg/ml horseradish peroxidase (Sigma Chem. Co.) in PBS for 2 h. The samples were further washed with 0.1 M PBS and were treated with 0.5 mg/ml 3,3′-diaminobenzidine (Sigma Chem. Co.) dissolved in PBS for color development and were finally treated with 6% hydrogen peroxide.

### Epith-2 isolation from ISO-DALT 2D gels

The location of the anti-Epith-2 mAb-immunopositive spot in the ISO-DALT 2D gel was estimated by immunoblotting. Using ISO-DALT 2D gel aliquots, the estimated anti-Epith-2 mAb-positive spot was cut using a razor blade and was placed in tubes installed in elusion tank of Centriluter (Amicon, Bevery, MA, USA) that was filled with SDS-PAGE tank buffer (25 mM Tris, 192 mM glycine, 0.1% SDS). Epith-2 was eluted from the gels at 4°C for 4 h at 200 V according to the manufacturer’s instructions. The elution buffer was replaced with 0.1 M PBS, the samples were concentrated using Centricon YM-10 tubes (Amicon) and were subsequently mixed with an equal volume of 2× non-reducing sample buffer for analysis with SDS-PAGE.

### Digestion of Epith-2 with trypsin and chymotrypsin

The lyophilized *T. hardwicki* swimming blastulae were dissolved in trypsin digestion sample buffer (TdSB; 8 M urea, 50 mM Tris-HCl, 1 mM CaCl_2_, pH 8.0) at 2 mg/ml (sample solution). Trypsin or chymotrypsin (Sigma Chem. Co.) was diluted in TdSB at 2 mg/ml (trypsin or chymotrypsin solution; TCS). TCS was mixed with the sample solution at 1:1, 1:2, 1:10, and 1:25, incubated for 2 h at 37°C, and then diluted 1:1 in 2× SDS-PAGE sample buffer for immunoblotting with anti-Epith-2 mAb as described above. The sample solution without the enzyme was used as a negative control and was incubated for the same period as the solution containing the enzyme.

### Immunohistochemistry

The eggs before and 20 min after insemination as well as the embryos at the appropriate developmental stages described in the text were fixed in 4% paraformaldehyde in FSW, dehydrated in ethanol, and embedded in Polywax. Six-micrometer thick sections were examined as described previously by Katow and Komazaki (27). Briefly, dewaxed Polywax sections were blocked with 5% BSA in 0.1 M PBS for 1 h, incubated with anti-Epith-2 mAb diluted 1:100 in PBS for 1 h, and washed with 0.1 M PBS three times (10 min each). These primary antibodies were visualized with goat tetramethylrhodamine-5-(and 6)-isothiocyanate (TRITC)-tagged anti-mouse IgG Ab (Promega; 1:300 diluted in 0.1 M PBS). The samples were embedded in glycerine, examined under an Optiphot fluorescence microscope (Nikon, Tokyo), and photographed with a CAMEDIA C-3030Z digital camera (Olympus Corporation, Tokyo).

For whole-mount immunohistochemistry, the fertilization envelopes of fertilized eggs and embryos before hatching were removed to allow antibody access as previously described (28). The eggs and embryos at the specific developmental stages were fixed in 4% paraformaldehyde, dehydrated in 70% ethanol, and stored at 4°C until use. Before primary Ab application, the stored embryos were hydrated in 0.1 M PBS with 1% Tween-20 (v/v) (PBST), blocked with 1% BSA in 0.1 M PBST for 1 h, and incubated with lyophilized mouse anti-Epith-2 mAb (1:10 in 0.1 M PBST) for 48 h at 4°C. Then, the samples were washed three times with 0.1 M PBST (10 min each). The primary Ab was visualized with Alexa Fluor 488-tagged rabbit anti-mouse IgG Ab (Invitrogen, 1:500 in 0.1 M PBST) for 2 h at ambient temperature. After washing three times with 0.1 M PBST (10 min each), the samples were examined under a Micro Radiance 2000 confocal laser scanning microscope (Bio-Rad, Hercules, CA, USA) with occasional optical sections at 2–4 μm, and the images were analyzed using an ImageJ 1.43u (NIH) and Photoshop CS5 Extended (Adobe Systems Inc., San Jose, CA, USA).

### PMC isolation

To examine whether cytoplasmic Epith-2 is modified from its state on the cell surface, PMCs in the blastocoel were isolated from *T. hardwicki* mesenchyme blastulae. Initially, PMCs were separated from the epithelial cells in calcium-magnesium-deprived artificial seawater (CMDASW; 463 mM NaCl, 11 mM KCl, 2.15 mM NaHCO_3_, 25.5 mM Na_2_SO_4_). The embryos were incubated in CMDASW for 10 min to remove the hyaline layer, precipitated by centrifugation at 1000 rpm for 10 s, and the discarded supernatant was replaced with fresh CMDASW. The embryos were gently pipetted to dissociate to single cells that were fixed with 4% paraformaldehyde diluted in CMDASW for 1 h, washed with 0.1 M PBST three times (10 min each), and incubated with anti-P4 mAb diluted 1:100 in 0.1 M PBST for 20 min at 4°C. The primary Ab was washed as describe above and incubated with magnetic Microbead-tagged MACS goat anti-mouse IgG Ab (Miltenyi Biotec GmbH, Bergisch Gladbach, Germany) diluted to 1:5 in PBST for 30 min at ambient temperature for magnetic cell separation. To visually test PMC purity, TRITC-tagged goat anti-mouse IgG Ab (diluted 1:300 in 0.1 M PBST) was also applied simultaneously to the magnetic Microbead-tagged secondary Ab. After secondary Ab treatment, the labeled PMCs were diluted at 2 × 10^8^ cells/ml and were applied to the magnetic cell separation column that was set in a MiniMACS cell separation apparatus (Miltenyi Biotec GmbH) according to the manufacturer’s instructions. The column was washed once with 0.1 M PBST that contains 0.5% BSA and 2 mM EDTA, and the column was then dismantled to obtain purified PMCs. The purity of the PMCs was examined under an Optiphot fluorescence microscope (Nikon, Tokyo). The purified PMC aliquots were further dissolved in SDS-sample buffer under reducing conditions and were analyzed by immunoblotting as described above. The PMC separation was summarized in a flowchart as shown in Figure 5F.

### Herbimycin A treatment

Early swimming blastulae were treated with 10 μg/ml herbimycin A (HA), a PTK inhibitor [Ref. (29–31): Wako Pure Chemical Co., Osaka, Japan], until the mesenchyme blastula stage, and then they were fixed and embedded in Polywax (32). The samples were sectioned, probed with anti-Epith-2 mAb, and examined by immunofluorescence microscopy as described above.

Aliquots of embryos were also used for immunoblotting after ISO-DALT 2D gel electrophoresis using anti-Epith-2 mAb, rabbit anti-phosphotyrosine (PT) Ab, rabbit anti-phosphoserine (PS) Ab, and rabbit anti-phosphothreonine (PY) Ab (Promega) as described above.

### Suramin treatment

Suramin, a GFR inhibitor [(33–35): Calbiochem Merck KGaA, Darstadt, Germany], was diluted in FSW at 150 mM and was applied to swimming blastulae to the stage when control embryos reached the early gastrula stage. Next, the embryos were fixed in 4% paraformaldehyde for whole-mount immunohistochemistry with anti-Epith-2 mAb and were then examined as described above.

### Embryonic cell reaggregation assay

To examine the potential involvement of Epith-2 in epithelial cell adhesion, anti-Epith-2 mAb IgG in conditioned medium was concentrated 30-fold using Centriprep 10 (Amicon), centrifuged for 10 min at 10,000 × *g* to remove precipitates, and IgG was purified using the HiTrap Protein G affinity column (Amersham Pharmacia Biotech, Buckinghamshire, UK). Thirty milliliters of conditioned medium produced 480 μg IgG.

The dissociated *H. pulcherrimus* swimming blastulae in CMDASW were washed once with fresh CMDASW and diluted at 1.6 × 10^6^ cells/ml. The anti-Epith-2 mAb IgG was added to the cell suspension in 24-well plates at 10 or 50 μg/ml and was placed in an incubator at 15°C for 5 h. The cell suspensions were examined under a light field microscope and photographed. The major axis of randomly chosen 80 cell aggregates was measured manually using printouts of micrographs of each sample, and the statistical significance was examined between the averages of two subjects using the unpaired *t* test by an online GraphPad software, QuickCalcs^1^ as shown in Figures 7E,F.

## Results

### Immunocrossreactivity of anti-Epith-1 and -2 mAbs among sea urchins

**Figure 1:**
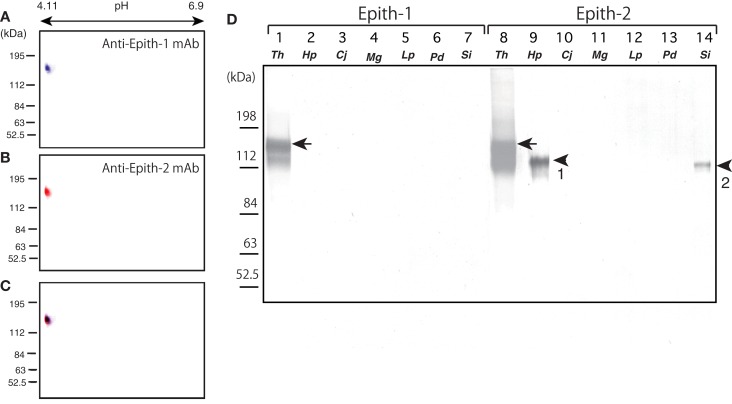
**Immunochemical property of antigen of anti-Epith-1 mAb and -2 mAb in *T. hardwicki* using isoelectric points and molecular mass in Daltons separation (ISO-DALT) 2D immunoblotting and immunocrossreactivity of these two mAbs among seven species of sea urchins**. **(A)** ISO-DALT 2D immunoblotting with anti-Epith-1 mAb shows an immunopositive spot at 160 kDa region and pH 4.98 (blue). The spot was artificially colored. **(B)** ISO-DALT 2D immunoblotting with anti-Epith-2 mAb shows an immunopositive spot (red) at the same region as **(A)**. The spot was artificially colored. **(C)** Merged image between **(A,B)**. **(D)** Immunocrossreactivity of anti-Epith-1 mAb (lanes 1–7) and anti-Epith-2 mAb (lanes 8–14). Lanes 1, 8; *T. hardwicki* (*Th*) swimming blastulae, lanes 2, 9; *H. pulcherrimus* (*Hp*) swimming blastulae, lanes 3, 10; *C. japonicus* (*Cj*) swimming blastulae, lanes 4, 11; *M. globules* (*Mg*) swimming blastulae, lanes 5, 12; *L. pictus* (*Lp*) swimming blastulae, lanes 6, 13; *P. depressus* (*Pd*) gastrulae, lanes 7, 14; *S. intermedius* mesenchyme blastulae (*Si*). Arrows, 160 kDa region. Arrowhead-1, 143 kDa region. Arrowhead-2, 137 kDa region.

Prior to onset of immunocrossreactivity assay of anti-Epith-1 mAb and -2 mAb in several species of the sea urchins as will be described below, immunochemical property of antigens of these mAbs was examined by ISO-DALT immunoblotting using *T. hardwicki*. The ISO-DALT immunoblotting showed both mAbs bound to a spot at 160 kDa and pH 4.98 region (Figures 1A–C), which is, regarding these two mAbs were raised as sister mAbs, predictable result.

Anti-Epith-1 and -2 mAbs were applied to *T. hardwicki* (Figure 1B, lanes 1, 8), *H. pulcherrimus* (Figure 1B, lanes 2, 9), *C. japonicus* (Figure 1B, lanes 3, 10), *M. globules* (Figure 1B, lanes 4, 11), and *L. pictus* swimming blastulae (Figure 1, lanes 5, 12), *P. depressus* gastrulae (Figure 1, lanes 6, 13), and *S. intermedius* mesenchyme blastulae (Figure 1B, lanes 7, 14). Anti-Epith-1 mAb bound only to *T. hardwicki* at the 160 kDa region, whereas anti-Epith-2 mAb bound to *T. hardwicki*, *H. pulcherrimus*, and *S. intermedius*, but not to the other four species examined in this study. The relative molecular mass (*M*_r_) of the anti-Epith-2 mAb-binding band of *H. pulcherrimus* and *S. intermedius* were slightly smaller than in *T. hardwicki* at 143 and 137 kDa, respectively. The relative intensity of the immunoreaction was also strongest in *T. hardwicki*, moderate in *H. pulcherrimus*, and weak in *S. intermedius*. This finding showed that the epitopic structures of the two mAbs were not identical but were similar to some extent among the sea urchins examined in the present study. Therefore, anti-Epith-1 mAb was highly specific to *T. hardwicki*, whereas anti-Epith-2 mAb crossreacted with a wider number of sea urchin species.

### Immunochemical property of Epith-2

**Figure 2:**
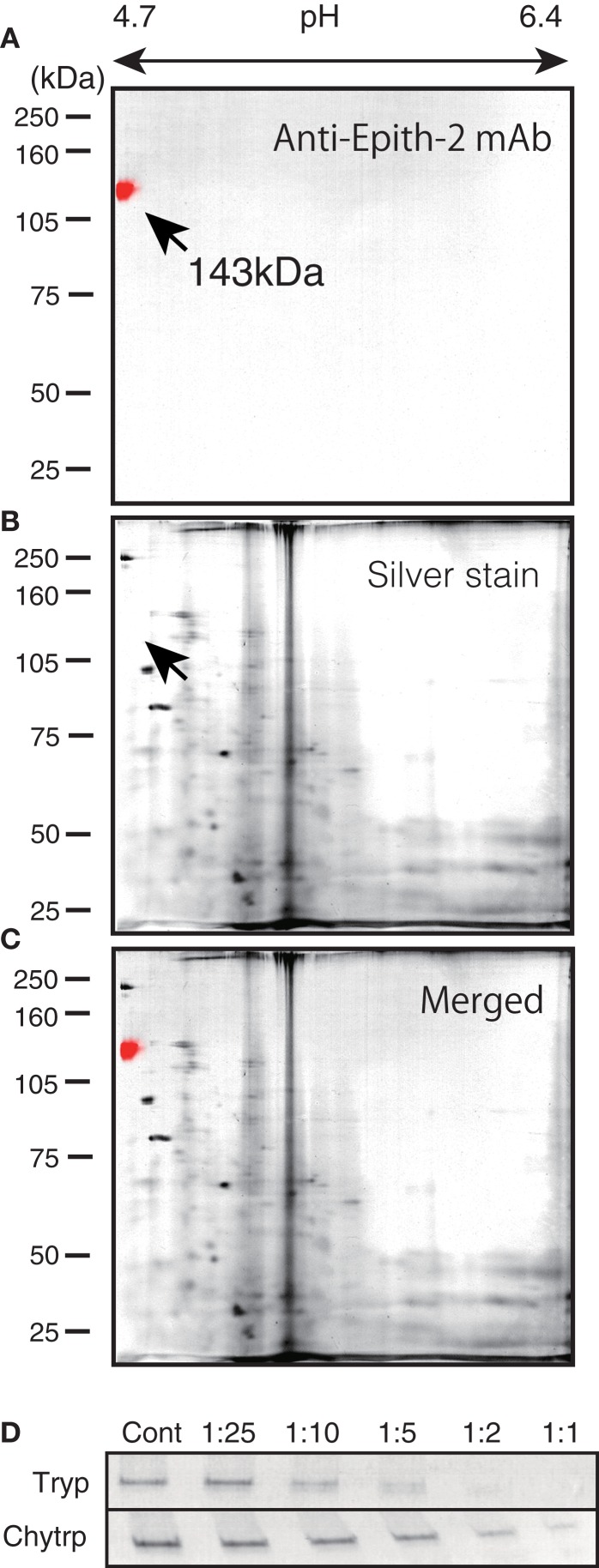
**Isoelectric points and molecular mass in Daltons separation (ISO-DALT) analysis of Epith-2 of *H. pulcherrimus* (A–C) and proteinase sensitivity of Epith-2 *T. hardwicki* (D)**. **(A)** ISO-DALT 2D immunoblotting shows an immunopositive spot at the 143 kDa and pH 4.7 (red) region. The spot is artificially colored. **(B)** ISO-DALT 2D pattern stained with silver. **(C)** Merged image between **(A,B)**. **(D)** Protease digestion of Epith-2 of swimming blastulae. Cont, control; the numbers, dilution ratios of protease, Tryp, trypsin digested. Chytrp, chymotrypsin digested.

To examine the similarities between the epitopes of anti-Epith-2 mAb of *H. pulcherrimus* and *T. hardwicki*, further proteomic analysis was conducted using ISO-DALT 2D immunoblotting using swimming blastulae. The immunoblotting localized an anti-Epith-2 mAb-binding spot at the acidic region [isoelectric point (*p*I) = 4.7; Figure 2A], which is similar to the previous report regarding *T. hardwicki* [*p*I = 4.98, Ref. (10)]. However, according to silver-stained ISO-DALT separation gel analysis, no positive spot was observed at the equivalent spot to the anti-Epith-2 mAb-binding spot region (Figure 2B, arrow), which was confirmed by a merged image (Figure 2C). In *T. hardwicki*, however, the 160 kDa region was weakly silver-stained using ISO-DALT 2D gel analysis (10). This weak stain may not be due to the small proportion of peptide but may be due to the highly acid property of Epith-2 (36).

The immunoblotting results using samples that were digested with trypsin and chymotrypsin indicated a decreased immunoreaction intensity according to the increasing concentration of both proteinases, which suggests that the anti-Epith-2 mAb epitope is located near the targets of these proteinases (Figure 2D). The sensitivity of the anti-Epith-2 mAb epitope to trypsin was apparently greater than the sensitivity to chymotrypsin, suggesting that the epitope is rich in lysine and/or arginine (targets of trypsin) compared to aromatic residues [target of chymotrypsin; Ref. (37)].

**Figure 3:**
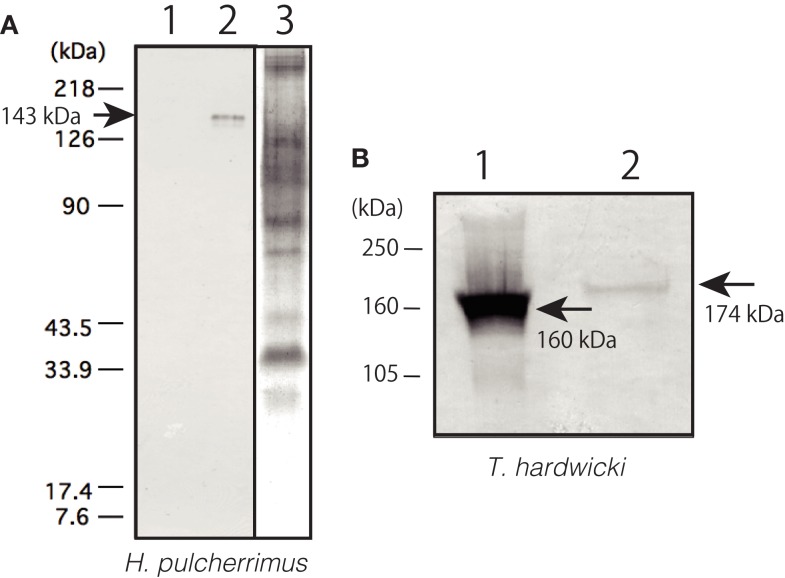
**Immunochemical property of Epith-2 of *H. pulcherrimu*s (A) and *T. hardwicki* (B) analyzed by immunoblotting**. **(A)** Immunoblotting of Epith-2 extracted in water (lane 1) and in non-ionic detergent 1% Triton X-100 (lane 2) of swimming blastulae. Whole embryo lysate stained with concanavalin A (lane 3). **(B)** *M_r_* of Epith-2 glycoprotein under non-reducing conditions (lane 1; 160 kDa region) and reducing conditions (lane 2; 174 kDa region).

The subcellular localization of Epith-2 was examined using lyophilized swimming blastulae of *H. pulcherrimus*. According to the immunoblotting, Epith-2 was not detected in the water-soluble fraction (Figure 3A, lane 1) but was detected in the non-ionic detergent-extract fraction at the 143 kDa region (Figure 3A, lane 2), which was consistent with previous Epith-1 analysis in *T. hardwicki* (10). The result suggested that Epith-2 is embedded in the plasma membrane or has a transmembrane domain (38). The Con-A staining of separated gels did not detect a 143 kDa band region (Figure 3A, lane 3), suggesting that unlike in the blastocoelar extracellular matrix in sea urchin embryos (26), Epith-2 has few or lacks mannose moieties.

To examine the potential presence of disulfide bonds in the *T. hardwicki* Epith-2 peptide, immunoblotting was conducted under reducing and non-reducing conditions. In non-reducing conditions, the anti-Epith-2 mAb strongly bound to a band at the 160 kDa region (Figure 3B, lane 1, arrow) but bound weakly to a band at the 174 kDa region under reducing conditions (Figure 3B, lane 2, arrowhead). Thus, Epith-2 was not found to have a subunit structure but was shown to have intra-peptide disulfide bonds that increased the *M*_r_ due to stretching of peptide chain under reducing conditions, which resulted in the spatial expansion of the peptide. The weakened immunoreactivity under reducing conditions suggests that the anti-Epith-2 mAb epitope located near the intra-peptide disulfide bonds region or to the region where the epitopic molecular configuration was easily affected by the loss of disulfide bonds.

### The Epith-2 expression pattern during early embryogenesis in *H. pulcherrimus*

**Figure 4:**
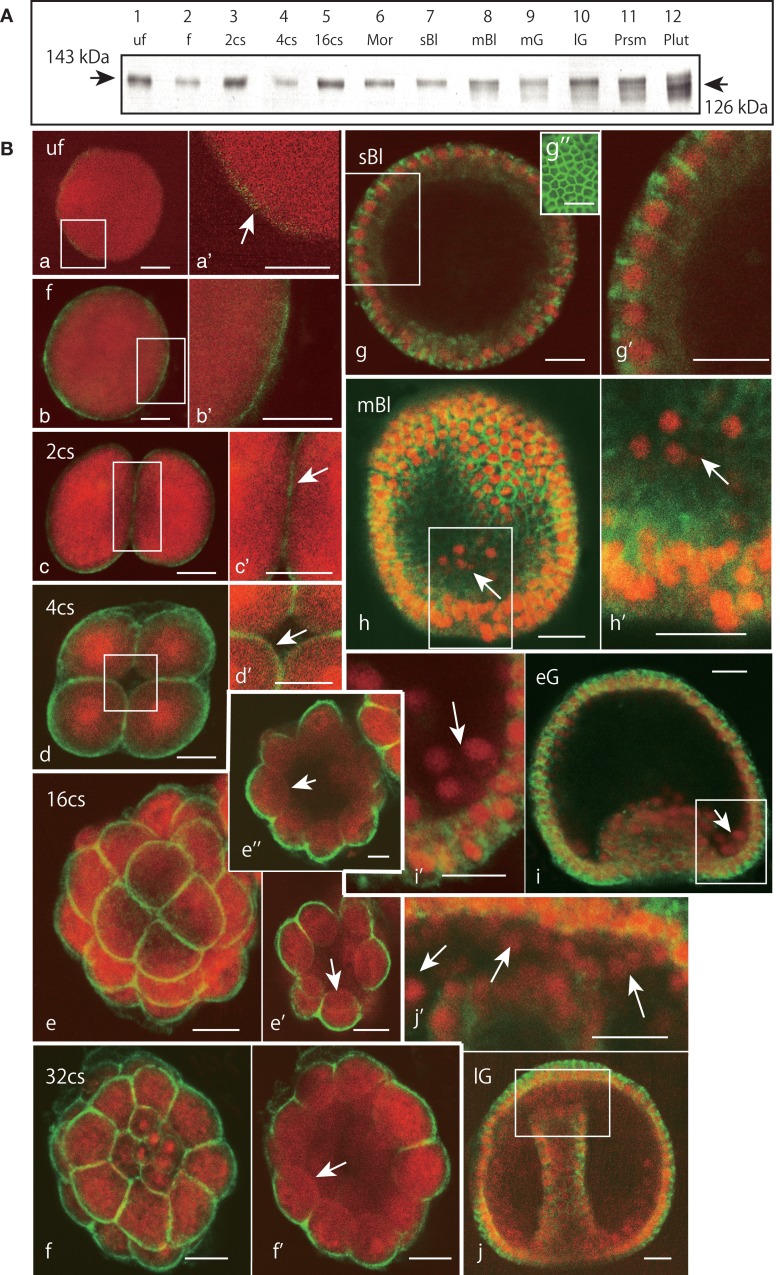
**Immunochemical expression pattern of Epith-2 during early development of *H. pulcherrimus***. **(A)** Immunoblotting pattern. Lane 1; unfertilized eggs (uf), lane 2; fertilized eggs (f), lane 3; the 2-cell stage embryos (2cs), lane 4; the 4-cell stage embryos (4cs), lane 5; the 16-cell stage embryos (16cs), lane 6; morulae (Mor), lane 7; swimming blastulae (sBl), lane 8; mesenchyme blastulae (mBl), lane 9; mid-gastrulae (mG), lane 10; late gastrulae (lG), lane 11; prism larvae (Prsm), lane 12; pluteus larvae (Plut). Epith-2 is detected consistently at the 143 kDa region from unfertilized eggs to prism larvae, but a new band is detected at the 126 kDa region at the pluteus stage, which replaced the larger band of relative molecular mass. **(B)** Whole-mount double-stained immunohistochemical expression pattern of Epith-2 (green) and DNA with propidium iodide (red) **(a–j’)** and Polywax sagittal 6-μm thick sections **(g”)**. **(a)** A 4-μm thick optical section of an unfertilized egg (uf). **(a’)** Higher magnification image of the box in **(a)**. Arrow, anti-Epith-2 mAb-positive egg surface. **(b)** A 4-μm thick optical section of a fertilized egg (f). **(b’)** Higher magnification image of the box in **(b)**. **(c)** A 4-μm thick optical section of a two-cell embryo (2cs). **(c’)** Higher magnification image of the box in **(c)**. **(d)** A 4-μm thick optical section of a 4-cell embryo (4cs). **(d’)** Higher magnification image of the box in **(d)**. Arrow, the basal surface of a blastomere. **(e)** Stacked image of a whole 16-cell stage embryo (16cs). **(e’)** A 3-μm thick optical section at the vegetal hemisphere. Arrow, the basal surface of a blastomere at the vegetal hemisphere. **(e”)** A 3-μm thick optical section at the animal hemisphere. Arrow, the basal surface of a blastomere. **(f)** Stacked image of a whole 32-cell embryos (32cs). **(f’)** A 3-μm thick optical section at the equator region. Arrow, the blastomere basal surface. **(g)** A 2-μm optical section of swimming blastula (sBl). **(g’)** Higher magnification image of the box in **(g)**. **(g”)** A 6-μm thick sagittal Polywax section. **(h)** A 2-μm thick optical section of a mesenchyme blastula (mBl). **(h’)** Higher magnification image of the box in **(h)**. Arrow, primary mesenchyme cells. **(i)** A 3-μm thick optical section of early mesenchyme blastula (eG). **(i’)** Higher magnification image of the box in **(i)**. Arrow, primary mesenchyme cells. **(j)** A 3-μm thick optical section of late gastrula (lG). **(j’)** Higher magnification image of the box in **(j)**. Arrows, secondary mesenchyme cells. Scale bars, 20 μm **(a–j’)**, 50 μm **(g”)**.

Based on the immunoblotting using the anti-Epith-2 mAb that was conducted with unfertilized eggs and embryos ranging to the 29-hpf prism stage, the protein was detected consistently at the 143 kDa region (Figure 4A, lanes 1–11). However, at the 40-hpf pluteus stage, the immunoreaction of the 143 kDa band weakened considerably. Instead, a smaller but distinctive new band appeared at the 126 kDa region (Figure 4A, lane 12), which is similar to our previous observations in *T. hardwicki* in which the major 160 kDa band shift to a smaller 142 kDa at this stage (10). This finding also suggests that the post-maternal message of the Epith-2 protein was switched on during the period between the 29- and 40-hpf larva stages as was previously reported regarding the Hp-Unc-5 expression pattern in *H. pulcherrimus* (39). However, further elucidative studies ought to be done.

Whole-mount immunohistochemistry faintly detected an anti-Epith-2 mAb-positive area near the egg surface in unfertilized eggs, which was not associated with any particular feature (Figures 4Ba,a’, arrow). In the eggs 20 min post fertilization, the mAb detected a thin but distinctively strong positive area near the egg surface (Figures 4Bb,b’). At the 2-hpf two-cell stage, the entire surface of the blastomeres was positive to the mAb that included the furrow region (Figures 4Bc,c’, arrow). At the 2.5-hpf 4-cell stage, the entire surface of all the blastomeres remained positive to the mAb, including the region that faces the newly formed blastocoel (Figures 4Bd,d’, arrow). After following two more cleavages at the 5.5-hpf 16-cell stage, while the entire apical surface of the blastomeres remained positive to anti-Epith-2 mAb (Figure 4Be), the basal surface around the vegetal pole area (Figure 4Be’, arrow) and the animal hemisphere (Figure 4Be”, arrow) lost Epith-2, which was the first sign of the apico-lateral distribution of Epith-2 in the embryogenesis. The apico-lateral distribution remained in the next 6.5-hpf 32-cell stage (Figures 4Bf,f’, arrow); however, at the 16-hpf swimming blastula stage, Epith-2 was lost from the apical surface of the epithelial cells (Figures 4Bg,g’). Epith-2 remained only on the lateral surface where the protein completely encircled the epithelial cells (Figure 4Bg”). At the 19-hpf mesenchyme blastula stage, Epith-2 was not detected on the entire surface of PMCs spread into the blastocoel (arrows in Figures 4Bh,h’) and in cellular aggregates at two sessile sites near the blastopore at the 22-hpf early gastrula stage (Figures 4Bi,i’). SMC that ingressed around the tip region of the archenteron also lost Epith-2 from their surface at the 25-hpf late gastrula stage (Figures 4Bj,j’, arrows). Thus, all the mesenchyme cells lost Epith-2 from their surface after ingression.

### The mechanism of cell surface Epith-2 retraction during mesenchyme ingression

**Figure 5:**
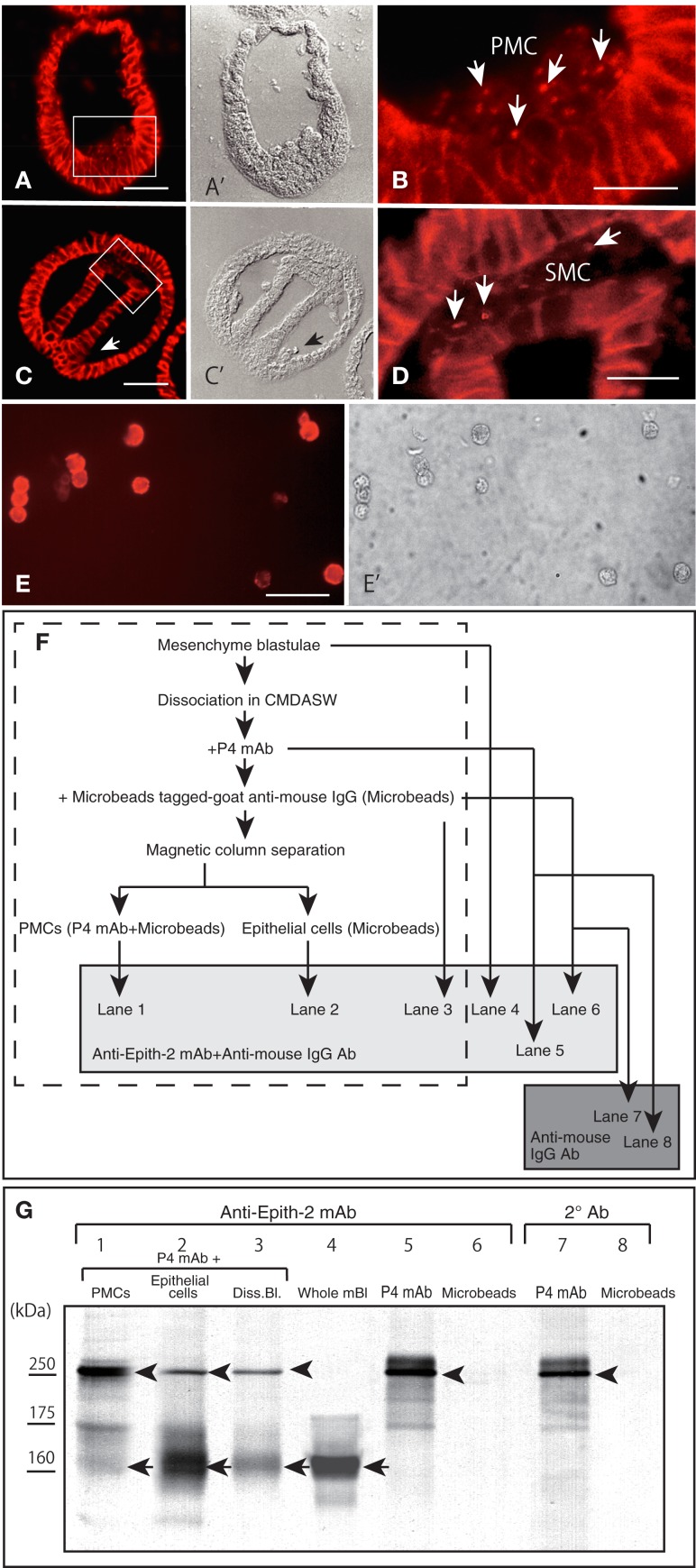
**Immunohistochemistry of Epith-2 internalization during mesenchyme ingression in *H. pulcherrimus* [(A–D); 6-μm thick Polywax sections], purified primary mesenchyme cells [PMC; (E,E’)] and the fate of Epith-2 in ingressed PMCs by immunoblotting in *T. hardwicki* (F,G)**. **(A)** An early mesenchyme blastula. Scale bar, 40 μm. **(A’)** Phase-contrast micrograph of the same section as **(A)**. **(B)** Higher magnification image of the box in **(A)**. Arrows, cytoplasmic anti-Epith-2 mAb-positive dots in primary mesenchyme cells (PMC). Scale bar, 10 μm. **(C)** Late gastrula. Scale bar, 40 μm. **(C’)** Phase-contrast micrograph of the same section as **(C)**. Arrow, PMC aggregate near the blastopore. **(D)** Higher magnification image of the box in **(C)**. Arrows, anti-Epith-2 mAb-positive dots in secondary mesenchyme cells (SMC). Scale bar, 10 μm. **(E)** Isolated PMCs stained with anti-P4 mAb. Scale bar, 30 μm. **(E’)** Phase-contrast micrograph of the same cells as **(E)**. **(F)** A chart showing the sample preparation of the immunoblotting shown in **(G)**. Samples in the broken-line box contain anti-P4 mAb and anti-mouse IgG Ab-tagged-magnetized Microbeads. Samples in the shaded box were examined with anti-Epith-2 mAb and anti-mouse IgG Ab. Samples in dark box were examined only with anti-mouse IgG Ab. **(G)** Epith-2 in the cytoplasm of PMCs analyzed with anti-Epith-2 mAb (lanes 1–6) and secondary antibody (Ab) alone (lanes 7, 8). Lane 1; anti-P4 mAb and anti-mouse IgG Ab-tagged-magnetized Microbeads-treated PMC fraction (PMCs), lane 2; anti-P4 mAb-treated epithelial cell fraction (Epithelial cells), lane 3; anti-P4 mAb-treated dissociated mesenchyme blastulae (Diss. Bl.), lane 4; whole mesenchyme blastulae (Whole mBl), lanes 5, 7; anti-P4 mAb alone (P4 mAb), lanes 6, 8; anti-mouse IgG Ab-tagged magnetic Microbeads alone (Microbeads). Arrows, Epith-2 at 160 kDa region. Arrowheads, IgG of primary Ab.

According to the immunohistochemistry performed using Polywax sections, PMCs lost Epith-2 from their surface at large while they are still in the vegetal ectoderm (Figures 5A,A’), and instead, several anti-Epith-2 mAb-positive dots were detected in the cytoplasm at the 19-hpf early mesenchyme blastula stage (Figure 5B, arrows), suggesting the occurrence of Epith-2 internalization. However, cytoplasmic immunoreaction in the PMCs was not detected by the 22-hpf early gastrula stage (Figures 5C,C’, arrow). In 25-hpf late gastrulae, ingression of the SMC also initiated at the tip of the archenteron. As in the PMCs, Epith-2 was not detected on the large surface area of the SMC (Figures 5C,C’); however, it was present in the cytoplasm and was associated with cytoplasmic dots (Figure 5D, arrows), suggesting the occurrence of Epith-2 internalization in SMC ingression.

To examine whether the internalization of the plasma membrane-bound Epith-2 occurs in association with any proteolysis, as has been previously reported regarding LvG-cadherin/β-catenin complex internalization in micromeres in the sea urchin (40), the potential decrease in the *M_r_* of Epith-2 was analyzed by immunoblotting using PMCs isolated from *T. hardwicki* mesenchyme blastulae immediately after ingression. According to immunocytochemistry using anti-P4 mAb, the proportion of PMCs isolated using magnetic cell sorting system was greater than 90% (Figures 5E,E’). The sample preparation for the analysis of cytoplasmic Epith-2 in PMCs using immunoblotting is summarized in Figure 5F. The immunoblotting showed three anti-Epith-2 mAb-positive major bands at approximately 250, 175, and 160 kDa regions in purified PMCs that were pre-incubated with anti-P4 mAb and magnetic Microbead-tagged MACS goat anti-mouse IgG Ab (Figure 5G, lane 1). After separation of the PMCs, the mesenchyme blastulae without PMCs showed two major bands at approximately 250 and 160 kDa regions. A band at the 175 kDa region was significantly weaker compared to the band observed in the PMCs (Figure 5G, lane 2). Swimming blastulae that were pre-incubated with anti-Epith-2 mAb showed two bands at approximately 250 and 160 kDa regions (Figure 5G, lane 3); however, before combining the two antibodies, the mesenchyme blastulae demonstrated only one band at 160 kDa region (Figure 5F, lane 4), as was previously shown (Figure 1, lane 8). This finding suggests that the 250 kDa band and, possibly, the 175 kDa band are derived from the anti-P4 mAb IgG, which has been confirmed by a following study of immunoblotting using the anti-P4 mAb alone, which showed a major band at 250 kDa region, a minor band at 175 kDa region and additional smaller minor bands but not at 160 kDa region (Figure 5G, lane 5). Because Microbead-tagged anti-mouse IgG Ab does not have anti-mouse IgG Ab or anti-goat IgG Ab epitopes, no immunoreaction was observed (Figure 5G, lane 6). Thus, any minor bands detected in the sample that was mixed with Microbead-tagged anti-mouse IgG Ab (Figure 5G, lanes 1–3) were not derived from the Ab. The immunoblotting procedure that applied secondary Ab alone to the anti-P4 mAb showed a major band at larger than 250 kDa region and weak smaller extra bands, indicating these bands were of degraded fragments of IgG (Figure 5G, lane 7). Again, Microbead-tagged anti-mouse IgG Ab did not show an immunoreaction (Figure 5G, lane 8).

Thus, an anti-Epith-2 mAb-positive band at 160 kDa region in the PMCs (Figure 5G, lane 1) is Epith-2, which suggests that because the cell surface Epith-2 *M*_*r*_ was retained in the cytoplasm, the protein was not cleaved appreciably during internalization and retained its molecular integrity after internalization.

### Inhibition of Epith-2 internalization by HA and suramin

The immunohistochemistry experiments conducted in this study suggest that the loss of Epith-2 from the PMC surface may be involved in PMC ingression and/or spreading.

The phosphorylation of tyrosine residues of SUp62, a cytoplasmic homo-dimeric 62 kDa protein, in PMCs that spread after ingression to the blastocoel occur in response to the contact of PMCs with pamlin, a blastocoelar PMC adhesion protein in the basal lamina (32, 41). HA inhibited PMC spreading in mesenchyme blastulae associated with hyperphosphorylation of tyrosine residues of SUp62 in *H. pulcherrimus* (32). In *T. hardwicki*, HA inhibited Epith-1 internalization along with P4 transportation to the cell surface of PMCs (23).

**Figure 6:**
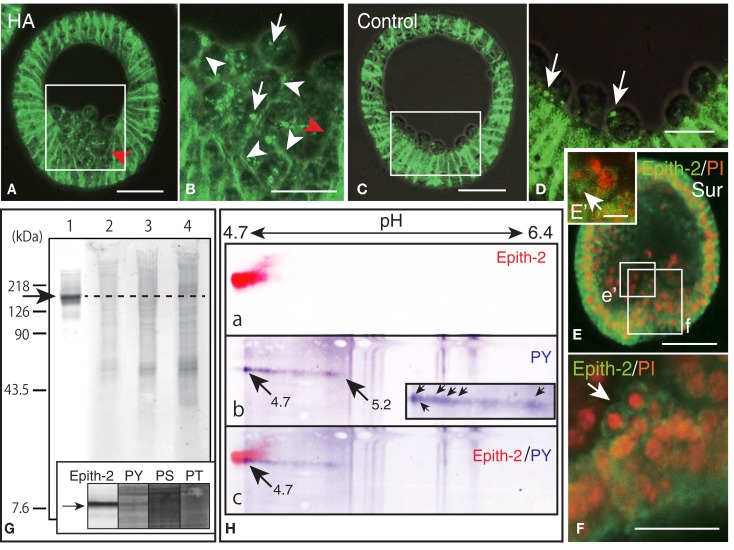
**Inhibition of Epith-2 endocytosis and PMC spreading by herbimycin A (HA) and suramin and the analysis of phosphorylation site of Epith-2 of *H. pulcherrimus***. **(A)** Immunohistochemistry of HA-treated mesenchyme blastula using 6-μm thick Polywax section. Red arrow, lack of Epith-2 on the cell surfaces of PMCs and neighboring epithelial cells. Scale bar, 40 μm. **(B)** Higher magnification of box in **(A)**. Arrowheads, cell-surface-associated Epith-2. Arrows, dots of cytoplasmic Epith-2. Red arrow, lack of Epith-2 on the cell surface of PMC and neighboring epithelial cell. Scale bar, 20 μm. **(C)** Immunohistochemistry of control mesenchyme blastula using 6-μm thick Polywax section. Scale bar, 40 μm. **(D)** Higher magnification of the box in **(C)**. Arrows, cytoplasmic Epith-2. Scale bar, 20 μm. (**E**) A 2-μm thick optical section of a confocal laser scanning micrograph of a suramin-treated early gastrula. Epith-2 expressed in an aggregate of PMCs near the archenteron [box **(e’)**]. Epith-2 (green) expressed in aggregated-PMCs on top of the archenteron [box **(f)**]. Scale bar, 40 μm. **(E’)** Higher magnification of the box **(e’)** in **(E)**. Arrow, Epith-2 cell. Scale bar, 5 μm **(F)** Higher magnification image of the box **(f)** in **(E)**. Arrow, aggregated PMCs. Scale bar, 20 μm. (**G**) Epith-2 phosphorylation in swimming blastulae by immunoblotting. Lane 1; anti-Epith-2 mAb, lane 2; anti-phosphotyrosine (PY) antibody (Ab), lane 3; anti-phosphoserine (PS) Ab, and lane 4; anti-phosphothreonine (PT) Ab. Arrow and dotted line denote the 143 kDa region. Inset; immunoblotting with antibodies against Epith-2, phosphotyrosine (PY), phosphoserine (PS), and phosphothreonine (PT) using single-lane SDS-PAGE gel. Arrow, 143 kDa region. **(H)** Phosphotyrosine detection by immunoblotting in Epith-2 of swimming blastulae by isoelectric points and molecular mass in Daltons. **(a)** Anti-Epith-2 mAb. Artificially colored with red. **(b)** Anti-PY Ab. Artificially colored with blue. Inset, higher magnification of tail region. Arrows, anti-PY Ab-positive dots. **(c)** Merged image between **(a,b)**. Numbers on the top show the pH gradient from the left (4.7) to right (6.4).

To examine whether the internalization of cell surface Epith-2 is also affected by HA in *H. pulcherrimus*, mesenchyme blastulae were treated with HA, and the Epith-2 expression pattern was analyzed. Consistent with mesenchyme blastulae of *T. hardwicki*, PMC dissociation from the vegetal epithelium of *H. pulcherrimus* was not inhibited by HA, but their spreading was inhibited (Figure 6A). However, the dissociated PMCs remained aggregated on the vegetal plate, and substantial PMCs retained cell surface Epith-2, particularly between neighboring PMCs (Figure 6B, arrowheads), while interestingly, peripheral complementary faces of PMCs that faced to epithelial cells (PE-border) lost their cell surface Epith-2 (Figures 6A,B, red arrowheads). In other than the PE-border region of the epithelium, cell surface Epith-2 remained expressed (Figure 6A). This finding suggests the occurrence of a centro-peripheral polarity of Epith-2 internalization in a group of PMCs, as well as the PE-border-restricted Epith-2 internalization in epithelium. In control blastulae, PMCs lost Epith-2 from their entire surface (Figures 4Bh–i, 5B, and 6C); however, a small number of PMCs retained cytoplasmic Epith-2 after spreading (Figure 6D). Thus, Epith-2 internalization appears to be involved in PMC dissociation from the epithelium and the spreading to single cells after ingression.

Primary mesenchyme cells spreading is also perturbed by suramin, which inhibits migration via the mitogen-activated protein kinase (MAPK) pathway (21). To examine whether PMC spreading occurs in relation to Epith-2 internalization, suramin was applied to swimming blastulae for 10 h until the early gastrula stage. In early gastrulae, consistent with our previous report (21), a characteristic aggregate of PMCs was observed on the archenteron tip during the early stage of invagination (Figure 6E). The aggregated PMCs retained Epith-2 on their cell surfaces (Figure 6F, arrow), indicating that Epith-2 internalization of these PMCs was inhibited by the GFR inhibitor. Other PMC aggregates near the archenteron in the blastocoel were also observed, and Epith-2 was also detected in these PMCs (Figure 6E’, arrow), which suggests the occurrence of migration associated with epithelial morphology after dissociation. Furthermore, spread cells were also observed in the blastocoel, but these cells did not display Epith-2 on their cell surface or in the cytoplasm (Figure 6E). Our previous report that these spread cells do not form spicules (21) suggests that they may not be PMCs. The suramin-treated embryos failed to complete gastrulation, and pigment cells were not formed (21). Thus, SMC ingression was not confirmed in this study.

Despite highly similar behavior between Epith-1 and Epith-2, whether the perturbation of internalization caused by the PTK inhibitor occurs in relation to phosphorylation of tyrosine, serine, and threonine residues of Epith-1 and Epith-2 has remained unanswered. In this study, an initial examination using immunoblotting detected no apparent anti-Epith-2 mAb-positive band at 143 kDa region (Figure 6G, lane 1). Furthermore, Abs against phosphotyrosine (Figure 6G, lane 2), phosphoserine (Figure 6G, lane 3), and phosphothreonine (Figure 6G, lane 4) also did not detect an anti-Epith-2 mAb-positive band at 143 kDa region. However, closer examination with an anti-phosphotyrosine Ab alone detected a positive band at the region quite similar to the anti-Epith-2 mAb-positive region (Figure 6G inset, lane PS).

To identify the molecules that bound to anti-phosphotyrosine Ab- and anti-Epith-2 mAb, ISO-DALT 2D immunoblotting analysis was conducted. Consistent with the above immunoblotting results obtained using slab gels, Epith-2 was localized at a region with *p*I and *M_r_* that were 4.7 and 143 kDa, respectively (Figure 6Ha). Phosphotyrosine also localized at 143 kDa and the near pH 4.7 region, but it had a stretched tail toward the basic region near pH 5.2 (Figure 6Hb, arrows). The tail comprised of a line of dots with the strongest immunoreaction at pH 4.7 region (Figure 6Hb, inset, arrows). Merged images localized the region that was positive to both anti-Epith-2 mAb and anti-phosphotyrosine Ab at approximately 143 kDa and *p*I = 4.7 region (Figure 6Hc, arrow). This finding suggests the occurrence of tyrosine phosphorylation in Epith-2.

### The potential role of Epith-2 in cell adhesion

The close association of Epith-2 internalization with mesenchyme cell ingression suggests that Epith-2 may be involved in cell–cell adhesion. Although Epith-1 association with cell-cell adhesion using anti-Epith-1 mAb in *T. hardwicki* was shown to be negative (10), based on the present result suggesting that the epitopic structure of anti-Epith-1 mAb and anti-Epith-2 mAb was different (Figure 1), the apparently close relationship between Epith-2 behavior with mesenchyme cell ingression suggested the potential involvement of the anti-Epith-2 mAb epitope in cell-cell adhesion.

**Figure 7:**
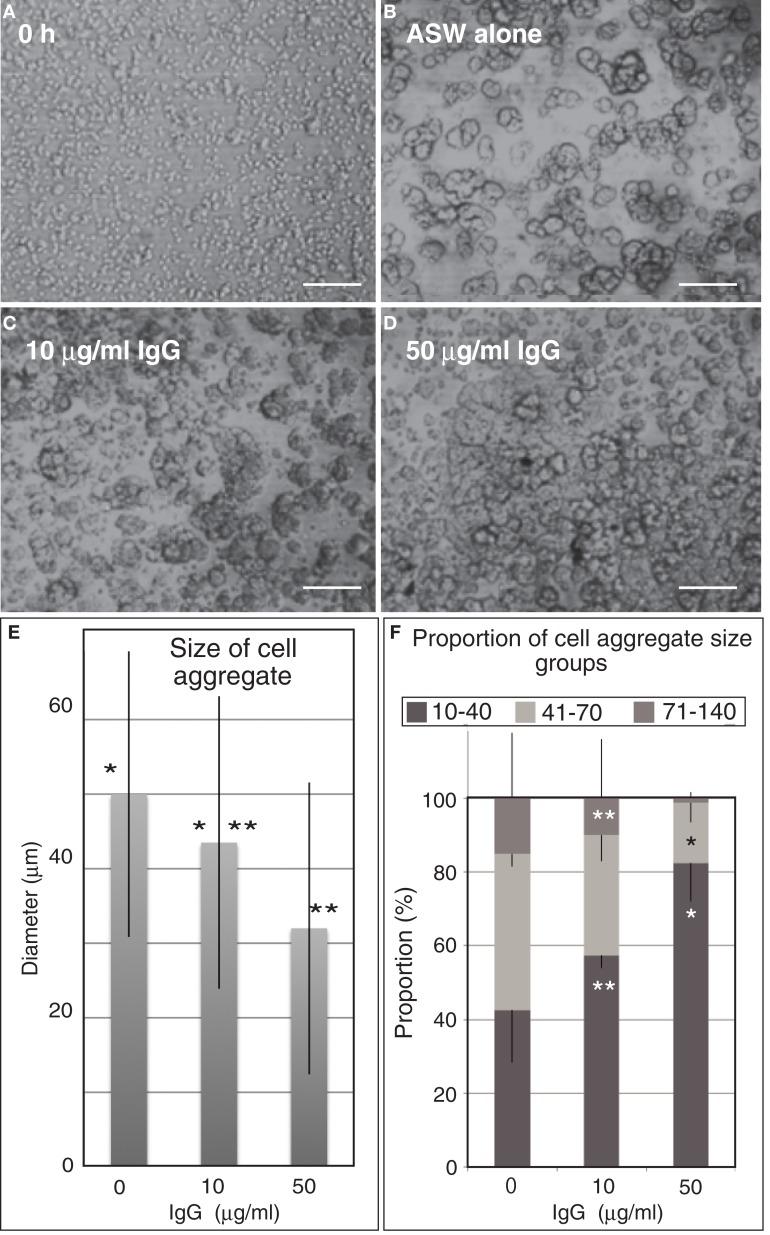
**Embryonic cell reaggregation assay in the presence of IgG of anti-Epith-2 monoclonal antibody in *H. pulcherrimu*s**. **(A)** Whole embryonic cells of swimming blastulae immediately after dissociation (0 h). **(B–D)** Re-aggregated embryonic cells at 5 h after dissociation. **(B)** In plain artificial seawater (ASW alone). **(C)** With 10 μg/ml IgG (10 μg/ml IgG). **(D)** With 50 μg/ml IgG (50 μg/ml IgG). Scale bars, 100 μm. **(E)** Average size of cell aggregates with no IgG (0), 10 μg/ml (10) and 50 μg/ml (50) IgG. Bars, SD (*n* = 80). *P = 0.0344, **P = 0.0001. Unpaired *t* test. **(F)** Proportion of three cell aggregate sizes in 0 μg/ml (0), 10 μg/ml (10), and 50 μg/ml (50) anti-Epith-2 mAb IgG. Bars, SD (*n* = 80). *P = 0.0055, **P = 0.0588. Unpaired *t* test.

To examine this possibility, the anti-Epith-2 mAb IgG was purified and applied to the dissociated embryonic cells *in vitro* to examine whether the epitope of the mAb interferes with the reaggregation of these cells. Dissociated embryonic cells (Figure 7A) re-aggregated in 5 h and formed large aggregates with various smaller sizes in ASW without IgG of anti-Epith-2 mAb (Figure 7B). The presence of 10 μg/ml of IgG did not visibly affect the size of the cell aggregates (Figure 7C). However, an apparent decrease in the size of the cell aggregates was observed in cells treated with 50 μg/ml of IgG (Figure 7D). To examine this morphological observation further, the major diameter of 80 cell aggregates chosen at random was measured in each experimental cell culture group. The average size of the cell aggregates decreased based on IgG treatment in a dose-dependent manner (10–50 μg/ml), which was found to be statistically significant between the control ASW groups (no IgG included) and the 10 μg/ml IgG-treated group (*P* = 0.0344) and between the 10 μg/ml IgG-treated and 50 μg/ml IgG-treated groups (*P* = 0.0001) (Figure 7E). According to the analysis of the proportion of the cell aggregates that were artificially sorted into three groups based on the size of the major axis of the aggregate, the group with the smallest aggregate size (10–40 μm) increased in size based on IgG treatment in a dose-dependent manner. However, the group with the largest size (71–140 μm) decreased based on IgG treatment in a dose-dependent manner; *P* = 0.0055 in 10 μg/ml IgG and *P* = 0.0231 in 50 μg/ml IgG (Figure 7F). In 50 μg/ml IgG-treated cell suspensions, the proportion of the medium-sized group (41–70 μm) was significantly smaller than that of the group with the smallest size (*P* = 0.0588). Thus, unlike the anti-Epith-1 mAb epitope, anti-Epith-2 mAb epitope was involved in cell–cell adhesion.

## Discussion

### Anti-Epith-1 mAb and anti-Epith-2 mAb epitopes

The anti-Epith-2 mAb was first generated in *T. hardwicki* as a sister mAb of anti-Epith-1 mAb. The mAb recognizes an Epith-1glycoprotein that contains *N*-glycosylated oligosaccharide, and the glycoprotein is present specifically in epithelial cells (10). Thus, the overall properties of the anti-Epith-2 mAb were similar to that of the anti-Epith-1 mAb as described in this study. However, the present study revealed a significantly different epitopic property of anti-Epith-2 mAb as well. The anti-Epith-2 mAb cross-reacts with two other sea urchin species, *H. pulcherrimus* and *S. intermedius*, in addition to *T. hardwicki*. Thus, although the evolutionary concept of Epith-2 is not the subject of the present study, the anti-Epith-2 mAb-binding epitope of these sea urchins that belong to Echinoidea and Temnopleuroida of Echinoidea (42) is similar, which may shed new insights into the evolutional significance of cell surface properties in future studies.

Although relevance of using intact divalent Abs against cell surface proteins in attempt to inhibit cell–cell adhesion is not generalized (43), consistent with previous reports (44–46), the present mAb utilization apparently worked. Thus, regardless whether the Ab is applied as an intact divalent form or a monovalent form, the validity would be dependent on their molecular property in antigen/Ab interaction.

The immunoreaction of Epith-2 to its mAb was significantly weakened by digestion with trypsin and not chymotrypsin, suggesting the presence of lysine and/or arginine-rich regions (L/R-rich region) in the peptide (37) near the anti-mAb epitope. Furthermore, the present cell reaggregation assay conducted in the presence of anti-Epith-2 mAb IgG indicates that the mAb epitope is located in the extracellular region of the protein and is involved in cell adhesion activity. The L/R-rich region of the protein may be involved in the cell adhesion activity of Epith-2. Interestingly the lysine-rich region is reported in the junction-associated protein, zonula occludens-1, in mammalian cells (47) and the arginine-rich region in vitronectin, a major cell adhesion protein (48). This region in vitronectin stimulates the activity of TGFβ (49). Further proteomic analysis will enable to determine the functional significance of the L/R-rich region of Epith-2 in the GFR signaling pathway.

### Endocytic retraction of Epith-2 from the surface of mesenchyme precursor cells during EMT

The present immunohistochemical detection of Epith-2 in the cytoplasm of mesenchyme cells and the observation of apparent GFR/PTK-dependent Epith-2 internalization suggest the involvement of growth factor(s). In *S. purpuratu*s, an FGF receptor (SpFGFR1) possesses conserved tyrosine kinase domain, and the transcripts accumulate when mesenchyme cell ingression and gastrulation occurs (50). Thus, the present and the previous observations suggest that the cell surface modification is carried out by endocytosis. The presently shown non-ionic detergent solubility of Epith-2 suggests that the protein is anchored to the plasma membrane via a transmembrane domain. Thus, the endocytic retraction of Epith-2 from the cell surface is reminiscent of a similar retraction of the LvG-cadherin/β-catenin complex in the micromere specification in *L. variegatus* (19, 20). The complex was removed from the cell surface by endocytosis (51), which was followed by the cleavage of β-catenin from LvG-cadherin and movement into the nucleus (40). According to the NCBI database^2^ and proteomic analysis using an open proteomic database ExPASy Compute pI/Mw (SIB Swiss Institute of Bioinformatics)^3^, the calculated *M*_*r*_ of *H. pulcherrimus* β-catenin was approximately 89.9 kDa, and the *p*I was 5.72 (BAN 13547). The *M_r_* of LvG-cadherin (AAC06341.1) was 303 kDa, and the *p*I was 3.88, whereas the *M*_*r*_ of Epith-2 was 143 kDa, and the *p*I was 4.7. Thus, Epith-2 is different from both proteins. Nevertheless, it is evident that the endocytic retraction of at least two cell surface proteins, Epith-2 and LvG-cadherin, are involved in the EMT and the acquisition of the mesenchymal property during and/or at the end of the EMT in sea urchin embryos.

The present isolated Epith-2 immunoblotting experiments of the ingressed PMCs indicated no detectable decrease in the *M*_*r*_, suggesting the occurrence of little or no Epith-2 degradation following endocytosis; however, later in the PMCs that spread into the blastocoels, cytoplasmic Epith-2 was not detected by immunohistochemistry. Thus, at least during the early period after endocytosis, Epith-2 retained its initial molecular configuration, including the anti-Epith-2 mAb epitopic structure, which suggests the potential occurrence of the prolonged activation of the Epith-2-mediated signaling pathway by constituting “signaling endosomes” [rev. (52)]. The present study did not determine whether tyrosine residues of Epith-2 were dephosphorylated in the endosomes as has been previously reviewed by Kholodenko (53). There are two major mechanisms of internalization of cell surface proteins: clathrin-mediated endocytosis and clathrin-independent lipid-raft-dependent endocytosis, and this latter mechanism includes the TGFβ receptor pathways [rev. (54)]. TGFβ contributes to numerous morphogenetic processes in sea urchin embryos (55, 56). According to immunoelectron microscopy images of bovine kidney epithelium, endocytosed cadherin was found to be associated with the zonula adherens plaque and the attached actin filaments in the juxtanuclear cytoplasm (57). Using *in vitro* astrocytes, immunoprecipitation studies indicated that neural cell adhesion molecules are endocytosed via a clathrin-dependent pathway (58). The endocytosis of DM-GRASP, a cell adhesion molecule of the immunoglobulin superfamily that promotes the growth and the navigation of axons in chick embryos, is dependent on clathrin. Immunoprecipitation studies have shown that internalized DM-GRASP enters the degradation pathway after ubiquitination with one or two ubiquitin(s) (59). Thus, substantial cell adhesion molecules are endocytosed via a clathrin-dependent pathway. However, whether Epith-2 is endocytosed via the clathrin pathway needs to be further addressed by immunoprecipitation experiments that will also clarify whether Epith-2 is ubiquitinated during immunochemical disappearance during the early gastrula stage as observed in PMCs. Whether LvG-cadherin is digested in the cytoplasm remains currently unknown.

The sea urchin genome contains at least 20 receptor tyrosine kinase genes, and among these genes, the accumulation of the FGFR1 transcripts is localized at the PMC and the SMC around the swimming blastula stage and the late gastrula stage, respectively, in *S. purpuratus* (56). This finding suggests the involvement of FGFR1 in mesenchyme differentiation or more precisely the ingression process. Tyrosine kinase induces endocytosis in bovine adrenal chromaffin cells (24), and gp60 induces endocytosis in pulmonary micro-vascular endothelial cells (60).

The apparent GFR/PTK-dependent Epith-2 endocytosis suggests the presence of an upstream growth factor(s), which is consistently reported in the vast EMT. TGFβ is known to be involved in the vast EMT (61–63) along with HGF, FGF, and EGF [rev. (64)], which is initiated by Notch signaling (3). The source of the growth factors in the sea urchin embryo [rev. (22)] and in vast animals (65, 66) is the extracellular matrix. The sea urchin genome contains numerous TGFβ (56), and its involvement in PMC skeletogenesis has been reported (67). TGFβ activates Ras (68), which is required for cell dissociation and spreading during the EGF-induced EMT of the *in vitro* NBT-II rat carcinoma cell line. The overexpression of the activated forms of c-Raf and MEK1 leads to cell dissociation. Consistently, the MEK1 inhibitor PD98059 inhibits EGF- and Ras-induced cell dispersion (69). Likewise, in *H. pulcherrimus*, PD98059 delays PMC ingression and inhibits SMC ingression as well as inhibits the phosphorylation of ERK that comprises a factor in the MAPK pathway (21). Thus, the MAPK pathway is essentially involved in mesenchyme cell dispersion or spreading. The phosphorylation by ERK activates the transcription factor Ets1 that is required for PMC specification (70), suggesting that endocytic internalization of Epith-2 initiates the activation of genes that are involved in the acquisition of mesenchymal properties. TGFβ also upregulates Snail (71), which plays an essential role in the EMT accompanied with the downregulation of E-cadherin and the upregulation of N-cadherin, known as “cadherin switch” in cancer metastasis [rev. (64)]. Likewise, in the sea urchin embryo, Snail is required for the endocytosis of cadherin downstream of Pmar1 and Alx1 through several downstream PMC-expressed proteins (72).

### Three-step process of mesenchyme ingression

Herbimycin A and suramin differently affected the ingression process of the mesenchyme. HA moderately inhibited, whereas suramin severely inhibited Epith-2 endocytosis. In particular, PMCs not only retained their epithelial morphology but also migrated after dissociation en masse, which caused PMCs to undergo “collective migration” as observed in several cancer cells that maintain cell–cell adhesions and epithelial morphology during metastasis (64). PMCs that undergo “collective migration” appear to support the present observation that Epith-2 retracted at the PE-border, while Epith-2 remained on the cell surface among PMCs. This finding suggests the occurrence of a centro-peripheral polarity in cell surface Epith-2 endocytic retraction during the early stage of ingression. Likewise, during the “collective migration” of several cancer cells such as L-10 human rectal adenocarcinoma cells, the cellular properties are different depending on the location of each cell (73). Thus, the centro-peripheral polarity of Epith-2 expression or endocytosis in the ingressed PMCs suggests that the GFR/PTK pathway is not involved in Epith-2 endocytosis at the PE-border. Further several control studies would further ensure the present inhibitory effects of HA and suramin.

Along with the present observation of the normal ingression process, the inhibition of EMT by HA and suramin suggest that at least three steps of the mesenchyme ingression process relates to GFR/PTK signaling: (1) GFR/PTK-dependent endocytic retraction of cell surface Epith-2; (2) GFR/PTK-independent dissociation from the epithelium; and (3) GFR-independent/TPK-dependent spreading to single cells and the acquisition of cell motility.

### Post-endocytosis of cell adhesion molecules

The endocytosis of cell surface adhesion proteins in the EMT results in the sole dissolution of the epithelial phenotype and in the activation of new sets of mesenchyme-specific genes. In sea urchin embryos, LvG-cadherin/β-catenin endocytosis triggers the activation of a substantial number of PMC-specific genes, including *Hp-ets* (74), *pmar1, alx1*, *ets1*, and *tbr* (72, 75). Based on perturbation studies, *pmar1* and *alx1* are positioned at upstream of *snail*, and their activation is a prerequisite of β-catenin endocytosis (72). In addition, PMC-specific gene activation cannot be regulated by the cell adhesion molecules. In Madin–Darby canine kidney cells, TGFβ1 induces *snail* expression, which triggers the EMT via a MAPK-dependent mechanism (76). However, conversely in breast cancer, Snail appears to activate the TGFβ pathway with Slug by increasing histone acetylation at the promoter region of TGFβ and the inhibition of the signaling pathway consequently decreases cell migration with no impact on cell junction molecules by Snail and Slug. These observations propose a dual regulatory system in the EMT: (1) the repression of the cell junction and (2) the cell migration through TGFβ and/or other pathways (77), which appears comparable to the present hypothesis suggesting a three-step process of mesenchyme ingression.

SMC-specific genes, such as *glial cells missing* (*gcm*), the *polyketide synthase* gene cluster (*pks-gc*), the *flavin-containing monooxygenases* multigene family (*fmo*), and *sulfotransferase* (*sult*), are isolated from the pigment cell lineage, and they are downstream of the Notch signaling pathway (78). Several of these genes may also be activated after endocytic retraction of the cell surface LvG-cadherin/β-catenin complex (19, 20) and, possibly, Epith-2.

## Conflict of Interest Statement

The authors declare that the research was conducted in the absence of any commercial or financial relationships that could be construed as a potential conflict of interest.

## Footnotes

^1^http://www.graphpad.com/quickcalcs/ttest1.cfm

^2^http://www.ncbi.nlm.nih.gov/

^3^http://web.expasy.org/compute_pi/

## References

[1] Viebahn C Mayer B Miething A Morphology of incipient mesoderm formation in the rabbit embryo: a light- and retrospective electron-microscopic study. Acta Anat (1995) 154:99–110 10.1159/000147756 8722509

[2] Hall BK The Neural Crest in Development and Evolution. New York: Springer (1999).

[3] Timmerman LA Grego-Bessa J Raya A Bertran E Perez-Pomares JM Diez J Notch promotes epithelial-mesenchymal transition during cardiac development and oncogenic transformation. Genes Dev (2004) 18:99–115 10.1101/gad.276304 14701881 PMC314285

[4] Kong D Li Y Wang Z Sarkar FH Cancer stem cells and epithelial-to-mesenchymal transition (EMT)-phenotypic cells: are they cousins or twins? Cancers (2011) 3:716–29 10.3390/cancers30100716 21643534 PMC3106306

[5] Joglekar MV Joglekar VM Joglekar SV Hardikar AA Human fetal pancreatic insulin-producing cells proliferate *in vitro*. J Endocrinol (2009) 201:27–36 10.1677/JOE-08-0497 19171567

[6] Schwanzel-Fukuda M Origin and migration of luteinizing hormone-releasing hormone neurons in mammals. Microsc Res Tech (1999) 44:2–10 10.1002/(SICI)1097-0029(19990101)44:1<2::AID-JEMT2>3.0.CO;2-4 9915559

[7] Dan K Cyto-embryology of echinoderm and amphibian. In: Bourne GH Danielli JK editors. International Review of Cytology. (Vol. 6), New York: Academic Press (1960). p. 321–67 10.1016/s0074-7696(08)62751-5 13813920

[8] Okazaki K Normal development to metamorphosis. In: Czihak G editor. The Sea Urchin Embryo: Biochemistry and Morphogenesis. Berlin: Springer-Verlag (1975). p. 177–232

[9] Katow H Solursh M Ultrastructure of primary mesenchyme cell ingression in the sea urchin *Lytechinus pictus*. J Exp Zool (1980) 213:231–46 10.1002/jez.1402130211

[10] Kanoh K Aizu G Katow H Disappearance of an epithelial cell surface-specific glycoprotein (Epith-1) associated with epithelial-mesenchymal conversion in sea urchin embryogenesis. Dev Growth Differ (2001) 43:83–95 10.1046/j.1440-169x.2001.00548.x 11148454

[11] Hörstadius S The mechanics of sea urchin development, studied by operative methods. Biol Rev (1939) 14:132–79 10.1111/j.1469-185X.1939.tb00929.x

[12] Anstrom JA Chin JE Leaf DS Parks AL Raff RA Localization and expression of msp130, a primary mesenchyme lineage-specific cell surface protein of the sea urchin embryo. Development (1987) 101:255–65 3128442 10.1242/dev.101.2.255

[13] Shimizu K Noro N Matsuda R Micromere differentiation in the sea urchin embryo: expression of primary mesenchyme cell specific antigen during development. Dev Growth Differ (1988) 30:35–47 10.1111/j.1440-169X.1988.00035.x 37282097

[14] Shimizu K Katow H Matsuda R Micromere differentiation in the sea urchin embryo: immunochemical characterization of primary mesenchyme cell-specific antigen and its biological role. Dev Growth Differ (1990) 32:629–36 10.1111/j.1440-169X.1990.00629.x 37281449

[15] Katow H Role of a primary mesenchyme cell surface specific antigen during early morphogenesis in sea urchin embryos. In: Yanagisawa K Yasumasu I Oguro C Suzuki N Motokawa T editors. Biology of Echinodermata. Rotterdam: Balkema (1991). p. 453–9

[16] Hertzler PL McClay DR AlphaSU2, an epithelial integrin that binds laminin in the sea urchin embryo. Dev Biol (1999) 207:1–13 10.1006/dbio.1998.9165 10049560

[17] Chagraoui J Lepage-Noll A Anjo A Uzan G Charbord P Fetal liver stroma consists of cells in epithelial-to-mesenchymal transition. Blood (2003) 101:2973–82 10.1182/blood-2002-05-1341 12506029

[18] Shimizu Y Yamamichi N Saitoh K Watanabe A Ito T Yamamichi-Nishina M Kinetics of v-src-induced epithelial-mesenchymal transition in developing glandular stomach. Oncogene (2003) 22:884–93 10.1038/sj.onc.1206174 12584568

[19] Miller JR McClay DR Changes in the pattern of adherens junction-associated β-catenin accompany morphogenesis in the sea urchin embryo. Dev Biol (1997) 192:310–22 10.1006/dbio.1997.8739 9441670

[20] Miller JR McClay DR Characterization of the role of cadherin in regulating cell adhesion during sea urchin development. Dev Biol (1997) 192:323–39 10.1006/dbio.1997.8740 9441671

[21] Katow H Aizu G Essential role of growth factor receptor-mediated signal transduction through the mitogen-activated protein kinase pathway in early embryogenesis of the echinoderm. Dev Growth Differ (2002) 44:437–55 10.1046/j.1440-169X.2002.00657.x 12392577

[22] Katow H Extracellular signal transduction in sea urchin embryogenesis: from extracellular matrix to MAP kinase pathway. Curr Top Peptide Protein Res (2003) 5:149–60

[23] Wessel GM Katow H Regulation of the epithelial-to-mesenchymal transition in sea urchin embryos. In: Savagner P editor. Rise and Fall of Epithelial Phenotype: Concepts of Epithelia-Mesenchyme Transition Molecular Biology Intelligence Unit. New York: Eureka.com/Landes Bioscience, Kluwer Academic/Plenum Publishers (2005). p. 77–100

[24] Nucifora PG Fox AP Tyrosine phosphorylation regulates rapid endocytosis in adrenal chromaffin cells. J Neurosci (1999) 19:9739–46 10559383 10.1523/JNEUROSCI.19-22-09739.1999 PMC6782969

[25] Rickwood D Chambers JAA Spragg SP Two-dimentional gel electrophoresis. 2nd ed In: Hames BD Rickwood D editors. Gel Electrophoresis of Proteins: A Practical Approach. Oxford: IRL Press (1990). p. 217–72

[26] Katow H Solursh M In situ distribution of concanavalin A binding sites in mesenchyme blastulae and early gastrulae of the sea urchin *Lytechinus pictus*. Exp Cell Res (1982) 139:171–80 10.1016/0014-4827(82)90330-5 6282600

[27] Katow H Komazaki S Spatio-temporal expression of pamlin during early embryogenesis in sea urchin and importance of N-linked glycosylation for the glycoprotein function. Roux’s Arch Dev Biol (1996) 205:371–81 10.1007/BF00377217 28306088

[28] Katow H Suyemitsu T Ooka S Yaguchi J Jin-nai T Kuwahara I Development of a dopaminergic system in sea urchin embryos and larvae. J Exp Biol (2010) 213:2808–19 10.1242/jeb.042150 20675551

[29] Honma Y Okabe-Kado J Kasukabe T Hozumi M Kodama H Kijigaya S Herbimycin A, an inhibitor of tyrosine kinase, prolongs survival of mice inoculated with myeloid leukemia C1 cells with high expression of *v-abl* tyrosine kinase. Cancer Res (1992) 52:4017–20 1617678

[30] Turturro F Arnold MD Frist AY Pulford K Model of inhibition of the NPM-ALK kinase activity by herbimycin A. Clin Cancer Res (2002) 8:240–5 11801565

[31] Gudi S Huvar I White CR McKnight NL Dusserre N Boss GR Rapid activation of Ras by fluid flow is mediated by Gaq and Gbg subunits of heterotrimeric G proteins in human endothelial cells. Arterioscler Thromb Vasc Biol (2003) 23:994–1000 10.1161/01.ATV.0000073314.51987.84 12714438

[32] Katow H Washio M Pamlin-induced tyrosine phosphorylation of SUp62 protein in primary mesenchyme cells during early embryogenesis in the sea urchin, *Hemicentrotus pulcherrimus*. Dev Growth Differ (2000) 42:519–29 10.1046/j.1440-169x.2000.00533.x 11041493

[33] Coffey RJ Jr. Leof EB Shipley GD Moses HL Suramin inhibition of growth factor receptor binding and mitogenicity in AKR-2B cell. J Cell Physiol (1987) 132:143–8 10.1002/jcp.1041320120 3496343

[34] Zhang Y-L Keng Y-F Zhao Y Wu L Zhang Z-Y Suramin is an active site-directed, reversible, and tight-binding inhibitor of protein-tyrosine phosphatases. J Biol Chem (1998) 273:12281–7 10.1074/jbc.273.20.12281 9575179

[35] Nakata H Stimulation of extracellular signal-regulated kinase pathway by suramin with concomitant activation of DNA synthesis in cultured cells. J Pharmacol Exp Ther (2004) 308:744–53 10.1124/jpet.103.058230 14593092

[36] Allen RC Saravis CA Maurer HR Gel Electrophoresis and Isoelectric Focusing of Proteins. Selected Techniques. Berlin: Walter de Gruyter (1984).

[37] Vajda T Szabo T Specificity of trypsin and alpha-chymotrypsin towards neutral substrates. Acta Biochim Biophys Acad Sci Hung (1976) 11:287–94 1026004

[38] Mayor S Maxfield FR Insolubility and redistribution of GPI-anchored proteins at the cell surface after detergent treatment. Mol Biol Cell (1995) 6:929–44 7579703 10.1091/mbc.6.7.929 PMC301249

[39] Abe K Katow T Ooka S Katow H Unc-5/netrin-mediated axonal projection during larval serotonergic nervous system formation in the sea urchin, *Hemicentrotus pulcherrimus*. Int J Dev Biol (2013) 57:415–25 10.1387/ijdb.120256hk 23873373

[40] Logan CY Miller JR Ferkowicz MJ McClay DR Nuclear β-catenin is required to specify vegetal cell fates in the sea urchin embryo. Development (1999) 126:345–57 9847248 10.1242/dev.126.2.345

[41] Katow H Pamlin, a primary mesenchyme cell adhesion protein, in the basal lamina of the sea urchin embryo. Exp Cell Res (1995) 218:469–78 10.1006/excr.1995.1180 7796882

[42] Barnes RSK Calow P Olive PJW Galding DW Spicer JI The Invertebrates: A Synthesis. 3rd ed Tokyo: Blackwell Science KK (2001).

[43] Mao X Chol EJ Payne AS Disruption of desmosome assembly by monovalent human pemphigus vulgaris monoclonal antibodies. J Invest Dermatol (2009) 129:908–18 10.1038/jid.2008.339 19037235 PMC2743719

[44] Springer WR Baronde SH Cell adhesion molecules: detection with univalent second antibody. J Cell Biol (1980) 87:703–7 10.1083/jcb.87.3.703 6970200 PMC2110804

[45] Damsky CH Richa J Solter D Knudsen K Buck CA Identification and purification of a cell surface glycoprotein mediating intercellular adhesion in embryonic and adult tissue. Cell (1983) 34:455–66 10.1016/0092-8674(83)90379-3 6352050

[46] Springer WR Haywood-Reid PL Antibodies specific for gp40 inhibit cell-cell adhesion by cross-linking the protein on the surface of *Dictyostelium purpureum*. J Cell Biochem (1993) 53:85–97 10.1002/jcb.240530202 8227191

[47] Gottardi CJ Arpin M Fanning AS Louvard D The junction-associated protein, zonula occludens-1, localizes to the nucleus before the maturation and during the remodeling of cell-cell contacts. Proc Natl Acad Sci U S A (1996) 93:10779–84 10.1073/pnas.93.20.10779 8855257 PMC38232

[48] Kost C Stuber W Ehrlich HJ Pannekock H Preissner KT Mapping of binding sites for heparin, plasminogen activator inhibitor-1 and plasminogen to vitronectin’s heparin binding region reveals a novel vitronectin-dependent feedback mechanism for the control of plasmin formation. J Biol Chem (1992) 267:12098–105 1376317

[49] Ribeiro SMF Schultz-Cherry S Murphy-Ullrich JE Heparin-binding vitronectin up-regulates latent TGF-β production by bovine aortic endothelial cells. J Cell Sci (1995) 108:1553–61 7542256 10.1242/jcs.108.4.1553

[50] McCoon PE Angerer RC Angerer LM SpFGFR, a new member of the fibroblast growth factor receptor family, is developmentally regulated during early sea urchin development. J Biol Chem (1996) 271:20119–25 10.1074/jbc.271.33.20119 8702734

[51] Nelson WJ Regulation of cell–cell adhesion by the cadherin–catenin complex. Biochem Soc Trans (2008) 36:149–55 10.1042/BST0360149 18363555 PMC3368607

[52] Pálfy M Reményi A Korcsmáros T Endosomal crosstalk: meeting points for signaling pathways. Trends Cell Biol (2012) 22:447–56 10.1016/j.tcb.2012.06.004 22796207 PMC3430897

[53] Kholodenko BN Four-dimensional organization of protein kinase signaling cascades: the roles of diffusion, endocytosis and molecular motors. J Exp Biol (2003) 206:2073–82 10.1242/jeb.00298 12756289

[54] Le Roy C Wrana JL Clathrin- and non-clathrin mediated endocytic regulation of cell signaling. Nat Rev Mol Cell Biol (2005) 6:112–26 10.1038/nrm1571 15687999

[55] Flowers VL Courteau GR Poustka AJ Weng W Venuti JM Nodal/activin signaling establishes oral-aboral polarity in the early sea urchin embryo. Dev Dyn (2004) 231:727–40 10.1002/dvdy.20194 15517584

[56] Lapraz F Röttinger E Duboc V Range R Duloquin L Walton K RTK and TGF-β signaling pathways genes in the sea urchin genome. Dev Biol (2006) 300:132–52 10.1016/j.ydbio.2006.08.048 17084834 PMC12337106

[57] Kartenbeck J Schmelz M Franke WW Geiger B Endocytosis of junctional cadherins in bovine kidney epithelial (MDBK) cells cultured in low Ca^2+^ ion medium. J Cell Biol (1991) 113:881–92 10.1083/jcb.113.4.881 2026652 PMC2288996

[58] Miñana R Duran JM Tomas M Renau-Piqueras J Guerri C Neural cell adhesion molecule is endocytosed via a clathrin-dependent pathway. Eur J Neurosci (2001) 13:749–56 10.1046/j.0953-816x.2000.01439.x 11207809

[59] Thelen K Georg T Bertuch S Zelinal P Pollerberg GE Ubiquitination and endocytosis of cell adhesion molecule DM-GRASP regulate its cell surface presence and affect its role for axon navigation. J Biol Chem (2008) 283:32792–801 10.1074/jbc.M805896200 18790729

[60] Minshall RD Tiruppathi C Vogel SM Malik AB Vesicle formation and trafficking in endothelial cells and regulation of endothelial barrier function. Histochem Cell Biol (2002) 117:105–12 10.1007/s00418-001-0367-x 11935286

[61] Miettinen PJ Ebner R Lopez AR Derynck R TGF-beta induced transdifferentiation of mammary epithelial cells to mesenchymal cells: involvement of type I receptors. J Cell Biol (1994) 127:2021–36 10.1083/jcb.127.6.2021 7806579 PMC2120317

[62] Runyan R Heimark RL Camenisch TD Klewer SE Epithelial-mesenchymal transformation in the embryonic heart. In: Savagner P editor. Rise and Fall of Epithelial Phenotype: Concepts of Epithelia-Mesenchyme Transition Molecular Biology Intelligence Unit. New York: Eureka.com/Landes Bioscience, Kluwer Academic/Plenum Publishers (2005). p. 40–55

[63] Vignalis M-L Fafet P TGF-β dependent epithelial-mesenchymal transition. In: Savagner P editor. Rise and Fall of Epithelial Phenotype: Concepts of Epithelia-Mesenchyme Transition Molecular Biology Intelligence Unit. New York: Eureka.com/Landes Bioscience, Kluwer Academic/Plenum Publishers (2005). p. 236–44

[64] Kawauchi T Cell adhesion and its endocytic regulation in cell migration during neural development and cancer metastasis. Int J Mol Sci (2012) 13:4564–90 10.3390/ijms13044564 22605996 PMC3344232

[65] Macri L Silverstein D Clark RAF Growth factor binding to the pericellular matrix and its importance in tissue engineering. Adv Drug Deliv Rev (2007) 59:1366–81 10.1016/j.addr.2007.08.015 17916397

[66] Clark RAF Synergistic signaling from extracellular matrix-growth factor complex. J Invest Dermatol (2008) 128:1354–5 10.1038/jid.2008.75 18478010

[67] Zito F Costa C Sciarrino S Poma V Russo R Angerer LM Expression of *univin*, a TGF-β growth factor, requires ectoderm–ECM interaction and promotes skeletal growth in the sea urchin embryo. Dev Biol (2003) 264:217–27 10.1016/j.ydbio.2003.07.015 14623243

[68] Janda E Nevolo M Lehmann K Downward J Beug H Grieco M Raf plus TGFbeta-dependent EMT is initiated by endocytosis and lysosomal degradation of E-cadherin. Oncogene (2006) 25:7117–30 10.1038/sj.onc.1209701 16751808

[69] Edme N Downward J Thiery JP Boyer B Ras induces NBT-II epithelial cell scattering through the coordinate activities of Rac and MAPK pathways. J Cell Sci (2002) 115:2591–601 12045229 10.1242/jcs.115.12.2591

[70] Rottinger E Besnardeau L Lepage T A Raf/MEK/ERK signaling pathway is required for development of the sea urchin embryo micromere lineage through phosphorylation of the transcription factor Ets. Development (2004) 131:1075–87 10.1242/dev.01000 14973284

[71] Peinado H Quintanilla M Cano A Transforming growth factor β-1 induces snail transcription factor in epithelial cell lines: mechanisms for epithelial mesenchymal transitions. J Biol Chem (2003) 278:21113–23 10.1074/jbc.M211304200 12665527

[72] Wu SY McClay DR The Snail repressor is required for PMC ingression in the sea urchin embryo. Development (2007) 134:1061–70 10.1242/dev.02805 17287249 PMC3045531

[73] Nabeshima K Inoue T Shimao Y Okada Y Itoh Y Seiki M Front-cell-specific expression of membrane-type 1 matrix metalloproteinase and gelatinase a during cohort migration of colon carcinoma cells induced by hepatocyte growth factor/scatter factor. Cancer Res (2000) 60:3364–9 10910039

[74] Kurokawa D Kitajima T Mitsunaga-Nakatsubo K Amemiya S Shimada H Akasaka K HpEts, an ets-related transcription factor implicated in primary mesenchyme cell differentiation in the sea urchin embryo. Mech Dev (1999) 80:41–52 10.1016/S0925-4773(98)00192-0 10096062

[75] Rho HK McClay DR The control of *foxN2/3* expression in sea urchin embryos and its function in the skeletogenic gene regulatory network. Development (2011) 138:937–45 10.1242/dev.058396 21303847 PMC3035096

[76] Pierce KL Maudsley S Daaka Y Luttrell LM Lefkowitz RJ Role of endocytosis in the activation of the extracellular signal regulated kinase cascade by sequestering and non sequestering G protein coupled receptors. Proc Natl Acad Sci U S A (2000) 97:1489–94 10.1073/pnas.97.4.1489 10677489 PMC26461

[77] Dhasarathy A Phadke D Deepak Mav D Shah RR Wade PA The transcription factors snail and slug activate the transforming growth factor-beta signaling pathway in breast cancer. PLoS One (2011) 6:e26514 10.1371/journal.pone.0026514 22028892 PMC3197668

[78] Calestani C Rast JP Davidson EH Isolation of pigment cell specific genes in the sea urchin embryo by differential macroarray screening. Development (2003) 130:4587–96 10.1242/dev.00647 12925586

